# Bis{tris­[3-(2-pyrid­yl)pyrazole]manganese(II)} dodeca­molybdosilicate hexa­hydrate

**DOI:** 10.1107/S1600536810001492

**Published:** 2010-01-27

**Authors:** Bao-Hua Niu, Tao Li, Yan Xi, Su-Xia Wu

**Affiliations:** a College of Medicine, Henan University, Kaifeng 475003, People’s Republic of China

## Abstract

The title compound, [Mn(C_8_H_7_N_3_)_3_]_2_[SiMo_12_O_40_]·6H_2_O, consists of an [SiMo_12_O_40_]^4−^ heteropolyanion, lying on a centre of inversion, and a complex [Mn(C_8_H_7_N_3_)_3_]^4+^ cation. The Mn^II^ atom of the cation is hexa­coordinated in a distorted octa­hedral geometry by six N atoms from three chelating 3-(2-pyrid­yl)pyrazole ligands. In the heteropolyanion, the four O atoms of the tetra­hedral SiO_4_ group each half-occupy eight sites due to Si lying on the centre of inversion. N—H⋯O and O—H⋯O hydrogen bonding mediated by the water mol­ecules leads to a consolidation of the structure.

## Related literature

For background to polyoxometalates, see: Pope & Müller (1991 ▶). For polyoxometalates modified with amines, see: Zhang, Dou *et al.* (2009 ▶); Zhang, Wei, Shi *et al.* (2010*a* ▶,*b* ▶);  Zhang, Wei, Sun *et al.* (2009 ▶); Zhang, Wei, Zhu *et al.* (2010 ▶); Zhang, Yuan *et al.* (2010 ▶). For another dodeca­molybdosilicate, see: Wu *et al.* (2003 ▶). 
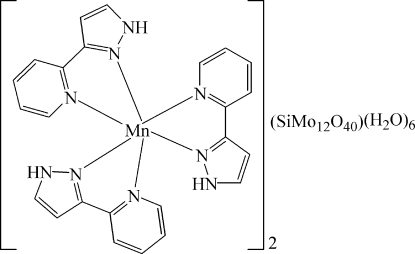


## Experimental

### 

#### Crystal data

[Mn(C_8_H_7_N_3_)_3_]_2_[SiMo_12_O_40_]·6H_2_O
*M* *_r_* = 2908.34
Monoclinic, 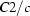

*a* = 18.907 (4) Å
*b* = 16.385 (3) Å
*c* = 27.552 (6) Å
β = 105.09 (3)°
*V* = 8241 (3) Å^3^
*Z* = 4
Mo *K*α radiation
μ = 2.17 mm^−1^
*T* = 293 K
0.12 × 0.10 × 0.08 mm

#### Data collection

Bruker APEXII CCD diffractometer
Absorption correction: multi-scan (*SADABS*; Bruker, 2001 ▶) *T*_min_ = 0.780, *T*_max_ = 0.845
27737 measured reflections
7045 independent reflections
5381 reflections with *I* > 2σ(*I*)
*R*_int_ = 0.054

#### Refinement

*R*[*F* ^2^ > 2σ(*F* ^2^)] = 0.059
*wR*(*F* ^2^) = 0.144
*S* = 1.01
7045 reflections
615 parameters
9 restraints
H atoms treated by a mixture of independent and constrained refinement
Δρ_max_ = 1.13 e Å^−3^
Δρ_min_ = −0.63 e Å^−3^

### 

Data collection: *APEX2* (Bruker, 2004 ▶); cell refinement: *SAINT-Plus* (Bruker, 2001 ▶); data reduction: *SAINT-Plus*; program(s) used to solve structure: *SHELXS97* (Sheldrick, 2008 ▶); program(s) used to refine structure: *SHELXL97* (Sheldrick, 2008 ▶); molecular graphics: *SHELXTL* (Sheldrick, 2008 ▶); software used to prepare material for publication: *SHELXTL*.

## Supplementary Material

Crystal structure: contains datablocks I, global. DOI: 10.1107/S1600536810001492/jh2123sup1.cif

Structure factors: contains datablocks I. DOI: 10.1107/S1600536810001492/jh2123Isup2.hkl

Additional supplementary materials:  crystallographic information; 3D view; checkCIF report

## Figures and Tables

**Table 1:** Hydrogen-bond geometry (Å, °)

*D*—H⋯*A*	*D*—H	H⋯*A*	*D*⋯*A*	*D*—H⋯*A*
N6—H6⋯O20	0.86	1.97	2.812 (12)	165
O1*W*—H1*W*⋯O2*W*	0.82 (5)	1.94 (3)	2.76 (3)	174 (12)
N9—H9*A*⋯O3*W*^i^	0.86	2.13	2.98 (2)	170
O3*W*—H5*W*⋯O3^ii^	0.82 (6)	2.16 (4)	2.937 (15)	157 (10)
O2*W*—H3*W*⋯O11^iii^	0.82 (13)	2.48 (12)	3.046 (19)	127 (12)
O2*W*—H3*WB*⋯O16^iv^	0.9 (6)	2.4 (4)	3.14 (2)	158

Symmetry codes: (i) 
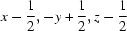
; (ii) 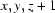; (iii) 
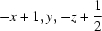
; (iv) 
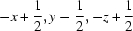
.

**Fig. 1.**
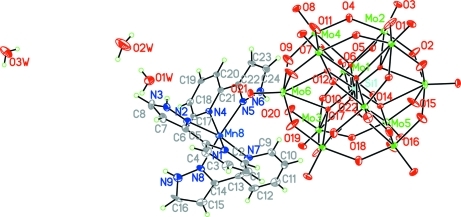
The cation and anion of the title compound with the atom-labelling scheme. Displacement ellipsoids are drawn at the 30% probability level; H atoms are given as spheres of arbitrary radius.

**Fig. 2.**
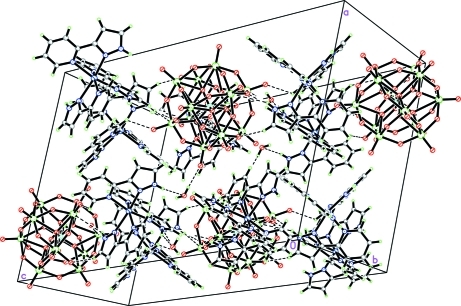
The crystal packing of the title compound, displayed with hydrogen bonds as dashed lines.

## supplementary crystallographic information

### Comment

There has been extensive interest in heteropolyoxometalates, owing to their fascinating properties and great potential applications in many fields (such as, catalysis, material science, medicine, and magnetochemistry) as well as their unusual topological properties (Pope *et al.*, 1991). The organic amines, such as 3-(2-pyridyl)pyrazole and pyrazine, are used to effectively modify heteropolyoxomolybdates under hydrothermal condictions (Zhang *et al.*, 2009*a,b*). Here, we describe the synthesis and structural characterization of the title compound.

As shown in Figure 1, the title compound consists of two subunits, *viz.* of a heteropolyanion [SiMo_12_O_40_]^4-^ anion, a complex [Mn(C_8_H_7_N_3_)_3_]^4+^ cation, and six lattice water molecules. The Mn^II^ ion is Hexa-coordinated in a distorted octahedron by six N atoms from three chelating 3-(2-pyridyl)pyrazole ligands. The Mn—N bond lengths are in the range of 2.207 (9)—2.272 (9) Å. In the Keggin structure anion, each Mo atom is surrounded by six O atoms and the Si atom is located at the center of the anion. There exists four kinds of O atoms according to their coordination environment: Oa (O atoms in the SiO4 trahedron), Ob (bridging O atoms between two triplet groups of MoO6 octahedra), Oc (bridging O atoms within one triplet group of MoO_6_ octahedra) and Od (terminal O atoms). The Si—O bond distances are in the normal range of 1.587 (2)—1.667 (2) compared to reported ones (Wu *et al.*, 2003). The Mo—O bond distances vary widely from 1.638 (10) to 2.444 (8) Å. The shortest Mo—O bonds are in the range of 1.638 (5)—1.665 (5) Å for the terminal oxygen atoms. The longest Mo—O lengths are in the range of 2.335 (3)—2.451 (8) Å for those oxygen atoms connected with both Mo and Si atoms. The Mo—O bond distances for the bridging oxygen atoms are from 1.809 (3) to 2.009 (1) Å. N—H···O and O—H···O hydrogen bonding between the neutral molecules and the water molecules leads to a consolidation of the structure (Fig. 2; Table 2).

### Experimental

A mixture of 3-(2-pyridyl)pyrazole (1 mmoL 0.14 g), sodium molybdate (2 mmoL, 0.48 g), sodium silicate nonahydrate (0.2 mmoL, 0.05 g) and Manganese sulfate monohydrate (1 mmoL, 0.17 g) in 10 ml distilled water was sealed in a 25 ml Teflon-lined stainless steel autoclave and was kept at 433 K for three days. Colorless crystals suitable for the X-ray experiment were obtained. Anal. Calc. for C_48_H_54_Mn_2_Mo_12_N_18_O_46_Si: C 19.81, H 1.86, N 8.67%; Found: C 19.65, H 1.72, N 8.55%.

### Refinement

All hydrogen atoms bound to carbon were refined using a riding model with distance C—H = 0.93 Å, *U*_iso_ = 1.2*U*_eq_ (C) for aromatic atoms. The H atoms of the water molecule were located from difference density maps and were refined with d(O—H) = 0.83 (2) Å, and with a fixed *U*_iso_ of 0.80 Å^2^. In the SiO_4_ unit, all oxygen atoms are disordered and their positions were refined with split positions and an occupancy ratio of 1:1. In the final difference Fourier map the highest peak is 2.29 Å from atom H2W and the deepest hole is 0.93 A Å from atom Mo6. The highest peak is located in the voids of the crystal structure and may be associated with an additional water molecule. However, refinement of this position did not result in a reasonable model. Hence this position was excluded from the final refinement.

### Crystal data

**Table:**

[Mn(C_8_H_7_N_3_)_3_]_2_[SiMo_12_O_40_]·6H_2_O	*F*(000) = 5616
*M**_r_* = 2908.34	*D*_x_ = 2.344 Mg m^−^^3^
Monoclinic, *C*2/*c*	Mo *K*α radiation, λ = 0.71073 Å
Hall symbol: -C 2yc	Cell parameters from 7045 reflections
*a* = 18.907 (4) Å	θ = 1.5–25.0°
*b* = 16.385 (3) Å	µ = 2.17 mm^−^^1^
*c* = 27.552 (6) Å	*T* = 293 K
β = 105.09 (3)°	Block, pink
*V* = 8241 (3) Å^3^	0.12 × 0.10 × 0.08 mm
*Z* = 4	

### Data collection

**Table:**

Bruker APEXII CCD diffractometer	7045 independent reflections
Radiation source: fine-focus sealed tube	5381 reflections with *I* > 2σ(*I*)
graphite	*R*_int_ = 0.054
phi and ω scans	θ_max_ = 25.0°, θ_min_ = 1.5°
Absorption correction: multi-scan (*SADABS*; Bruker, 2001)	*h* = −22→22
*T*_min_ = 0.780, *T*_max_ = 0.845	*k* = −19→19
27737 measured reflections	*l* = −32→32

### Refinement

**Table:**

Refinement on *F*^2^	Primary atom site location: structure-invariant direct methods
Least-squares matrix: full	Secondary atom site location: difference Fourier map
*R*[*F*^2^ > 2σ(*F*^2^)] = 0.059	Hydrogen site location: inferred from neighbouring sites
*wR*(*F*^2^) = 0.144	H atoms treated by a mixture of independent and constrained refinement
*S* = 1.01	*w* = 1/[σ^2^(*F*_o_^2^) + (0.060*P*)^2^ + 127.9076*P*] where *P* = (*F*_o_^2^ + 2*F*_c_^2^)/3
7045 reflections	(Δ/σ)_max_ = 0.001
615 parameters	Δρ_max_ = 1.13 e Å^−^^3^
9 restraints	Δρ_min_ = −0.63 e Å^−^^3^

### Special details

**Table:**

Geometry. All e.s.d.'s (except the e.s.d. in the dihedral angle between two l.s. planes) are estimated using the full covariance matrix. The cell e.s.d.'s are taken into account individually in the estimation of e.s.d.'s in distances, angles and torsion angles; correlations between e.s.d.'s in cell parameters are only used when they are defined by crystal symmetry. An approximate (isotropic) treatment of cell e.s.d.'s is used for estimating e.s.d.'s involving l.s. planes.
Refinement. Refinement of *F*^2^ against ALL reflections. The weighted *R*-factor *wR* and goodness of fit *S* are based on *F*^2^, conventional *R*-factors *R* are based on *F*, with *F* set to zero for negative *F*^2^. The threshold expression of *F*^2^ > σ(*F*^2^) is used only for calculating *R*-factors(gt) *etc*. and is not relevant to the choice of reflections for refinement. *R*-factors based on *F*^2^ are statistically about twice as large as those based on *F*, and *R*- factors based on ALL data will be even larger.

### Fractional atomic coordinates and isotropic or equivalent isotropic displacement parameters (Å^2^)

**Table:**

	*x*	*y*	*z*	*U*_iso_*/*U*_eq_	Occ. (<1)
Si1	0.2500	0.2500	0.0000	0.0238 (6)	
Mn8	0.20275 (8)	0.32148 (10)	0.30305 (6)	0.0499 (4)	
Mo1	0.35094 (4)	0.40389 (5)	0.07736 (3)	0.0410 (2)	
Mo2	0.41814 (4)	0.31787 (6)	−0.01901 (3)	0.0438 (2)	
Mo3	0.17562 (5)	0.34714 (6)	0.08913 (3)	0.0440 (2)	
Mo4	0.41640 (4)	0.20104 (6)	0.08819 (3)	0.0469 (2)	
Mo5	0.19369 (5)	0.45425 (5)	−0.01788 (4)	0.0468 (2)	
Mo6	0.24655 (6)	0.14836 (6)	0.11242 (3)	0.0511 (3)	
C1	0.0885 (6)	0.2487 (7)	0.2033 (5)	0.062 (3)	
H1	0.0835	0.3029	0.1930	0.075*	
C2	0.0494 (8)	0.1908 (11)	0.1719 (6)	0.088 (4)	
H2	0.0188	0.2053	0.1408	0.106*	
C3	0.0563 (8)	0.1110 (9)	0.1870 (6)	0.091 (5)	
H3	0.0309	0.0697	0.1666	0.109*	
C4	0.1015 (8)	0.0941 (9)	0.2331 (6)	0.088 (4)	
H4	0.1056	0.0406	0.2448	0.105*	
C5	0.1400 (6)	0.1527 (7)	0.2617 (4)	0.057 (3)	
C6	0.1915 (6)	0.1368 (7)	0.3104 (4)	0.054 (3)	
C7	0.2133 (8)	0.0644 (8)	0.3366 (5)	0.078 (4)	
H7	0.1954	0.0124	0.3269	0.094*	
C8	0.2654 (8)	0.0840 (8)	0.3787 (5)	0.080 (4)	
H8	0.2904	0.0483	0.4036	0.096*	
C9	0.1679 (7)	0.4862 (7)	0.2345 (5)	0.063 (3)	
H9	0.1976	0.4650	0.2155	0.075*	
C10	0.1422 (7)	0.5620 (8)	0.2259 (5)	0.068 (3)	
H10	0.1555	0.5930	0.2014	0.082*	
C11	0.0997 (9)	0.5939 (12)	0.2501 (7)	0.101 (5)	
H11	0.0821	0.6466	0.2426	0.122*	
C12	0.0815 (7)	0.5535 (10)	0.2844 (7)	0.090 (5)	
H12	0.0508	0.5764	0.3021	0.109*	
C13	0.1085 (6)	0.4732 (8)	0.2952 (5)	0.063 (3)	
C14	0.0871 (6)	0.4282 (8)	0.3333 (4)	0.059 (3)	
C15	0.0374 (8)	0.4441 (11)	0.3619 (6)	0.096 (5)	
H15	0.0074	0.4894	0.3607	0.115*	
C16	0.0447 (7)	0.3695 (14)	0.3953 (6)	0.113 (7)	
H16	0.0194	0.3582	0.4192	0.135*	
C17	0.2995 (6)	0.3995 (6)	0.4069 (4)	0.048 (2)	
H17	0.2567	0.3938	0.4174	0.057*	
C18	0.3587 (7)	0.4342 (7)	0.4391 (4)	0.060 (3)	
H18	0.3559	0.4531	0.4703	0.072*	
C19	0.4236 (6)	0.4412 (7)	0.4246 (4)	0.054 (3)	
H19	0.4651	0.4640	0.4460	0.065*	
C20	0.4246 (5)	0.4140 (7)	0.3785 (4)	0.054 (3)	
H20	0.4674	0.4182	0.3681	0.065*	
C21	0.3615 (5)	0.3793 (6)	0.3461 (4)	0.042 (2)	
C22	0.3585 (5)	0.3527 (6)	0.2960 (4)	0.045 (2)	
C23	0.4141 (6)	0.3462 (9)	0.2710 (4)	0.068 (3)	
H23	0.4635	0.3583	0.2835	0.082*	
C24	0.3795 (7)	0.3180 (9)	0.2241 (5)	0.072 (4)	
H24	0.4012	0.3088	0.1979	0.087*	
N1	0.1336 (5)	0.2310 (6)	0.2482 (4)	0.054 (2)	
N2	0.2303 (5)	0.1991 (6)	0.3359 (3)	0.056 (2)	
N3	0.2743 (5)	0.1662 (7)	0.3777 (4)	0.064 (3)	
N4	0.2995 (4)	0.3733 (5)	0.3613 (3)	0.0421 (19)	
N5	0.2962 (5)	0.3277 (5)	0.2663 (3)	0.051 (2)	
N6	0.3102 (5)	0.3063 (6)	0.2225 (3)	0.061 (3)	
H6	0.2780	0.2875	0.1970	0.073*	
N7	0.1515 (5)	0.4406 (6)	0.2700 (4)	0.064 (3)	
N8	0.1227 (5)	0.3571 (6)	0.3462 (4)	0.060 (3)	
N9	0.0967 (6)	0.3225 (8)	0.3825 (4)	0.084 (4)	
H9A	0.1111	0.2761	0.3960	0.101*	
O1	0.4018 (5)	0.2154 (5)	−0.0598 (4)	0.109 (4)	
O2	0.3538 (5)	0.3658 (5)	−0.0743 (4)	0.106 (4)	
O3	0.4979 (4)	0.3440 (5)	−0.0290 (3)	0.062 (2)	
O4	0.4530 (5)	0.2433 (5)	0.0359 (3)	0.071 (2)	
O5	0.4039 (5)	0.3952 (5)	0.0236 (3)	0.073 (2)	
O6	0.4010 (4)	0.4783 (5)	0.1107 (3)	0.066 (2)	
O7	0.4045 (5)	0.3127 (5)	0.1067 (3)	0.075 (2)	
O8	0.4923 (4)	0.1803 (5)	0.1310 (3)	0.069 (2)	
O9	0.3467 (5)	0.1770 (5)	0.1265 (4)	0.083 (3)	
O10	0.2789 (4)	0.3891 (6)	0.1087 (3)	0.076 (2)	
O11	0.3948 (5)	0.1018 (6)	0.0552 (4)	0.096 (4)	
O12	0.2946 (6)	0.1829 (6)	0.0405 (4)	0.028 (2)	0.50
O13	0.3293 (6)	0.2834 (6)	0.0270 (4)	0.025 (2)	0.50
O14	0.2846 (4)	0.4671 (6)	0.0256 (3)	0.083 (3)	
O15	0.2325 (5)	0.4385 (6)	−0.0712 (4)	0.103 (4)	
O16	0.1658 (4)	0.5493 (4)	−0.0260 (3)	0.068 (2)	
O17	0.2006 (6)	0.2371 (6)	0.0415 (4)	0.027 (2)	0.50
O18	0.1577 (4)	0.4246 (6)	0.0403 (3)	0.081 (3)	
O19	0.1482 (4)	0.3937 (5)	0.1343 (3)	0.063 (2)	
O20	0.2231 (5)	0.2542 (5)	0.1287 (3)	0.087 (3)	
O21	0.2460 (4)	0.0986 (5)	0.1638 (2)	0.059 (2)	
O22	0.2488 (6)	0.3354 (6)	0.0285 (4)	0.025 (2)	0.50
O1W	0.3845 (6)	0.2182 (8)	0.4579 (5)	0.114 (4)	
O2W	0.4625 (13)	0.0738 (17)	0.4742 (14)	0.292 (16)	
O3W	0.6311 (8)	0.3371 (13)	0.9351 (6)	0.195 (8)	
H1W	0.407 (2)	0.175 (2)	0.465 (4)	0.180*	
H2W	0.352 (5)	0.225 (6)	0.473 (4)	0.180*	
H3W	0.483 (7)	0.059 (9)	0.453 (4)	0.380*	
H3WB	0.420 (14)	0.07 (5)	0.48 (3)	0.380*	
H5W	0.590 (2)	0.348 (7)	0.938 (3)	0.280*	
H6W	0.636 (5)	0.343 (9)	0.9065 (17)	0.280*	
H3A	0.312 (3)	0.203 (4)	0.396 (4)	0.07 (4)*	

### Atomic displacement parameters (Å^2^)

**Table:**

	*U*^11^	*U*^22^	*U*^33^	*U*^12^	*U*^13^	*U*^23^
Si1	0.0229 (15)	0.0222 (16)	0.0245 (16)	−0.0009 (13)	0.0027 (12)	0.0004 (12)
Mn8	0.0404 (8)	0.0423 (9)	0.0661 (11)	−0.0054 (7)	0.0121 (8)	−0.0092 (8)
Mo1	0.0326 (4)	0.0433 (5)	0.0447 (5)	−0.0086 (4)	0.0061 (4)	−0.0126 (4)
Mo2	0.0284 (4)	0.0507 (5)	0.0529 (5)	−0.0092 (4)	0.0119 (4)	−0.0028 (4)
Mo3	0.0469 (5)	0.0486 (5)	0.0361 (5)	0.0103 (4)	0.0103 (4)	−0.0108 (4)
Mo4	0.0301 (4)	0.0578 (6)	0.0447 (5)	−0.0007 (4)	−0.0046 (4)	0.0053 (4)
Mo5	0.0527 (5)	0.0300 (4)	0.0614 (6)	0.0079 (4)	0.0214 (4)	0.0026 (4)
Mo6	0.0720 (6)	0.0474 (5)	0.0323 (5)	−0.0099 (5)	0.0104 (4)	0.0065 (4)
C1	0.056 (7)	0.057 (7)	0.066 (8)	0.000 (6)	0.002 (6)	−0.010 (6)
C2	0.075 (9)	0.111 (13)	0.072 (9)	0.009 (9)	0.006 (7)	−0.018 (9)
C3	0.090 (10)	0.068 (10)	0.102 (12)	−0.022 (8)	0.000 (9)	−0.038 (9)
C4	0.088 (8)	0.070 (7)	0.091 (8)	−0.016 (6)	−0.002 (6)	−0.009 (6)
C5	0.055 (7)	0.053 (7)	0.066 (7)	−0.010 (5)	0.019 (6)	−0.016 (6)
C6	0.063 (7)	0.045 (6)	0.060 (7)	−0.010 (5)	0.028 (6)	−0.005 (5)
C7	0.098 (10)	0.063 (9)	0.077 (9)	−0.015 (7)	0.029 (8)	0.000 (7)
C8	0.111 (12)	0.049 (8)	0.077 (9)	0.004 (7)	0.021 (8)	0.019 (7)
C9	0.073 (8)	0.050 (7)	0.073 (8)	−0.003 (6)	0.033 (7)	−0.018 (6)
C10	0.077 (7)	0.062 (7)	0.059 (6)	−0.004 (6)	0.007 (5)	0.000 (5)
C11	0.099 (9)	0.102 (9)	0.098 (9)	0.008 (7)	0.016 (7)	−0.019 (8)
C12	0.051 (8)	0.101 (12)	0.102 (12)	0.009 (8)	−0.009 (8)	−0.036 (10)
C13	0.041 (6)	0.062 (8)	0.076 (8)	0.009 (6)	−0.005 (6)	−0.025 (6)
C14	0.042 (5)	0.074 (7)	0.060 (6)	−0.003 (5)	0.009 (5)	−0.017 (5)
C15	0.072 (7)	0.112 (9)	0.097 (8)	0.009 (7)	0.010 (6)	−0.041 (7)
C16	0.041 (7)	0.23 (2)	0.075 (10)	−0.066 (10)	0.036 (7)	−0.070 (12)
C17	0.054 (6)	0.047 (6)	0.048 (6)	−0.007 (5)	0.021 (5)	−0.017 (5)
C18	0.077 (8)	0.047 (6)	0.049 (6)	0.015 (6)	0.003 (6)	−0.013 (5)
C19	0.050 (6)	0.058 (7)	0.042 (6)	0.014 (5)	−0.010 (5)	−0.011 (5)
C20	0.031 (5)	0.079 (8)	0.046 (6)	−0.001 (5)	−0.001 (4)	0.002 (6)
C21	0.040 (5)	0.038 (5)	0.048 (6)	0.003 (4)	0.011 (4)	0.002 (4)
C22	0.044 (6)	0.048 (6)	0.040 (5)	0.001 (5)	0.007 (4)	−0.002 (5)
C23	0.044 (6)	0.114 (11)	0.052 (7)	0.000 (7)	0.022 (5)	−0.016 (7)
C24	0.067 (8)	0.103 (11)	0.049 (7)	0.000 (7)	0.021 (6)	−0.004 (7)
N1	0.042 (5)	0.054 (6)	0.065 (6)	−0.007 (4)	0.010 (4)	−0.008 (5)
N2	0.050 (5)	0.054 (6)	0.061 (6)	−0.005 (4)	0.007 (5)	−0.002 (5)
N3	0.062 (6)	0.075 (7)	0.052 (6)	−0.014 (5)	0.012 (5)	0.002 (5)
N4	0.039 (4)	0.040 (5)	0.050 (5)	−0.007 (4)	0.016 (4)	−0.011 (4)
N5	0.046 (5)	0.062 (6)	0.041 (5)	0.000 (4)	0.005 (4)	−0.007 (4)
N6	0.066 (6)	0.079 (7)	0.033 (5)	0.004 (5)	0.005 (4)	−0.012 (5)
N7	0.042 (5)	0.056 (6)	0.089 (7)	0.000 (5)	0.009 (5)	−0.010 (6)
N8	0.041 (5)	0.073 (7)	0.071 (6)	−0.018 (5)	0.019 (5)	−0.016 (5)
N9	0.066 (7)	0.092 (9)	0.088 (8)	−0.042 (6)	0.008 (6)	−0.009 (7)
O1	0.101 (7)	0.039 (5)	0.129 (8)	0.015 (5)	−0.075 (6)	−0.013 (5)
O2	0.108 (7)	0.042 (5)	0.116 (8)	0.028 (5)	−0.066 (6)	−0.027 (5)
O3	0.048 (4)	0.063 (5)	0.088 (6)	−0.005 (4)	0.041 (4)	−0.003 (4)
O4	0.108 (6)	0.058 (4)	0.060 (4)	0.031 (4)	0.044 (4)	0.012 (4)
O5	0.107 (6)	0.062 (5)	0.061 (5)	0.042 (4)	0.043 (4)	0.013 (4)
O6	0.077 (5)	0.060 (5)	0.057 (5)	−0.024 (4)	0.013 (4)	−0.024 (4)
O7	0.111 (6)	0.065 (5)	0.068 (5)	0.023 (4)	0.058 (4)	0.010 (4)
O8	0.054 (5)	0.092 (6)	0.049 (5)	0.023 (4)	−0.007 (4)	−0.003 (4)
O9	0.091 (5)	0.083 (5)	0.095 (6)	−0.043 (4)	0.059 (5)	−0.042 (5)
O10	0.042 (4)	0.113 (6)	0.072 (5)	0.012 (4)	0.016 (4)	0.039 (5)
O11	0.103 (7)	0.113 (8)	0.099 (7)	−0.067 (6)	0.073 (6)	−0.068 (6)
O12	0.032 (6)	0.019 (6)	0.033 (6)	0.003 (5)	0.007 (5)	−0.008 (5)
O13	0.025 (6)	0.020 (6)	0.029 (6)	−0.006 (4)	0.008 (5)	0.002 (4)
O14	0.049 (4)	0.127 (7)	0.074 (5)	0.019 (4)	0.021 (4)	0.051 (5)
O15	0.081 (6)	0.133 (8)	0.126 (8)	−0.073 (6)	0.080 (6)	−0.085 (7)
O16	0.055 (5)	0.035 (4)	0.110 (7)	0.012 (3)	0.013 (4)	0.017 (4)
O17	0.022 (5)	0.030 (6)	0.024 (6)	0.000 (5)	0.000 (4)	0.003 (5)
O18	0.040 (4)	0.123 (6)	0.075 (5)	0.006 (4)	0.010 (4)	0.038 (5)
O19	0.050 (4)	0.087 (6)	0.056 (5)	−0.002 (4)	0.023 (4)	−0.030 (4)
O20	0.084 (5)	0.050 (4)	0.091 (6)	0.001 (4)	−0.040 (4)	−0.007 (4)
O21	0.072 (5)	0.071 (5)	0.034 (4)	−0.009 (4)	0.013 (3)	0.011 (4)
O22	0.031 (6)	0.026 (6)	0.020 (5)	−0.006 (5)	0.008 (5)	−0.005 (4)
O1W	0.079 (8)	0.144 (11)	0.106 (9)	−0.012 (7)	0.003 (6)	0.000 (8)
O2W	0.22 (2)	0.23 (2)	0.51 (5)	−0.002 (18)	0.26 (3)	0.08 (3)
O3W	0.207 (17)	0.226 (19)	0.208 (18)	−0.037 (15)	0.153 (15)	−0.054 (15)

### Geometric parameters (Å, °)

**Table:**

Si1—O13	1.587 (10)	C4—C5	1.332 (16)
Si1—O13^i^	1.587 (10)	C4—H4	0.9300
Si1—O22^i^	1.607 (10)	C5—N1	1.332 (14)
Si1—O22	1.607 (10)	C5—C6	1.462 (16)
Si1—O12^i^	1.635 (11)	C6—N2	1.343 (13)
Si1—O12	1.635 (11)	C6—C7	1.395 (17)
Si1—O17^i^	1.667 (11)	C7—C8	1.352 (18)
Si1—O17	1.667 (11)	C7—H7	0.9300
Mn8—N2	2.207 (9)	C8—N3	1.358 (15)
Mn8—N8	2.232 (9)	C8—H8	0.9300
Mn8—N5	2.255 (9)	C9—N7	1.330 (15)
Mn8—N7	2.262 (10)	C9—C10	1.333 (16)
Mn8—N4	2.262 (8)	C9—H9	0.9300
Mn8—N1	2.272 (9)	C10—C11	1.281 (19)
Mo1—O6	1.665 (7)	C10—H10	0.9300
Mo1—O10	1.809 (7)	C11—C12	1.27 (2)
Mo1—O7	1.866 (8)	C11—H11	0.9300
Mo1—O14	1.935 (8)	C12—C13	1.414 (19)
Mo1—O5	1.998 (7)	C12—H12	0.9300
Mo1—O22	2.334 (10)	C13—N7	1.312 (15)
Mo1—O13	2.386 (10)	C13—C14	1.426 (18)
Mo2—O3	1.659 (7)	C14—N8	1.347 (15)
Mo2—O5	1.794 (7)	C14—C15	1.398 (19)
Mo2—O2	1.858 (8)	C15—C16	1.51 (2)
Mo2—O4	1.922 (8)	C15—H15	0.9300
Mo2—O1	1.999 (8)	C16—N9	1.364 (19)
Mo2—O17^i^	2.348 (10)	C16—H16	0.9300
Mo2—O13	2.421 (10)	C17—N4	1.328 (12)
Mo3—O19	1.655 (7)	C17—C18	1.359 (15)
Mo3—O1^i^	1.797 (8)	C17—H17	0.9300
Mo3—O18	1.816 (8)	C18—C19	1.390 (16)
Mo3—O20	1.950 (8)	C18—H18	0.9300
Mo3—O10	2.007 (8)	C19—C20	1.349 (14)
Mo3—O17	2.349 (11)	C19—H19	0.9300
Mo3—O22	2.439 (10)	C20—C21	1.410 (13)
Mo4—O8	1.639 (7)	C20—H20	0.9300
Mo4—O11	1.856 (8)	C21—N4	1.348 (11)
Mo4—O4	1.886 (8)	C21—C22	1.435 (13)
Mo4—O7	1.928 (8)	C22—N5	1.313 (12)
Mo4—O9	1.932 (8)	C22—C23	1.403 (14)
Mo4—O12	2.355 (10)	C23—C24	1.367 (16)
Mo4—O13	2.434 (10)	C23—H23	0.9300
Mo5—O16	1.641 (7)	C24—N6	1.315 (15)
Mo5—O15	1.825 (8)	C24—H24	0.9300
Mo5—O14	1.834 (8)	N2—N3	1.344 (12)
Mo5—O11^i^	1.951 (8)	N3—H3A	0.97 (8)
Mo5—O18	1.960 (8)	N5—N6	1.346 (11)
Mo5—O12^i^	2.359 (10)	N6—H6	0.8600
Mo5—O22	2.413 (11)	N8—N9	1.348 (14)
Mo6—O21	1.637 (7)	N9—H9A	0.8600
Mo6—O20	1.873 (8)	O1—Mo3^i^	1.797 (8)
Mo6—O9	1.890 (8)	O2—Mo6^i^	1.929 (8)
Mo6—O2^i^	1.929 (8)	O11—Mo5^i^	1.951 (8)
Mo6—O15^i^	1.925 (8)	O12—Mo5^i^	2.359 (10)
Mo6—O17	2.409 (10)	O13—O22	1.753 (15)
Mo6—O12	2.451 (11)	O15—Mo6^i^	1.925 (8)
C1—N1	1.340 (14)	O17—Mo2^i^	2.348 (10)
C1—C2	1.364 (17)	O1W—H1W	0.82 (5)
C1—H1	0.9300	O1W—H2W	0.83 (10)
C2—C3	1.37 (2)	O2W—H3W	0.82 (13)
C2—H2	0.9300	O2W—H3WB	0.9 (6)
C3—C4	1.361 (19)	O3W—H5W	0.82 (6)
C3—H3	0.9300	O3W—H6W	0.82 (7)
			
O13—Si1—O13^i^	180.0 (12)	O21—Mo6—O17	157.0 (4)
O13—Si1—O22^i^	113.4 (5)	O20—Mo6—O17	65.0 (4)
O13^i^—Si1—O22^i^	66.6 (5)	O9—Mo6—O17	98.5 (4)
O13—Si1—O22	66.6 (5)	O2^i^—Mo6—O17	62.3 (4)
O13^i^—Si1—O22	113.4 (5)	O15^i^—Mo6—O17	93.1 (5)
O22^i^—Si1—O22	180.0 (9)	O21—Mo6—O12	154.4 (4)
O13—Si1—O12^i^	110.2 (5)	O20—Mo6—O12	98.0 (4)
O13^i^—Si1—O12^i^	69.8 (5)	O9—Mo6—O12	63.1 (4)
O22^i^—Si1—O12^i^	108.7 (5)	O2^i^—Mo6—O12	96.1 (4)
O22—Si1—O12^i^	71.3 (5)	O15^i^—Mo6—O12	61.2 (4)
O13—Si1—O12	69.8 (5)	O17—Mo6—O12	48.4 (4)
O13^i^—Si1—O12	110.2 (5)	N1—C1—C2	123.1 (12)
O22^i^—Si1—O12	71.3 (5)	N1—C1—H1	118.5
O22—Si1—O12	108.7 (5)	C2—C1—H1	118.5
O12^i^—Si1—O12	180.0 (12)	C1—C2—C3	118.5 (14)
O13—Si1—O17^i^	69.5 (5)	C1—C2—H2	120.7
O13^i^—Si1—O17^i^	110.5 (5)	C3—C2—H2	120.7
O22^i^—Si1—O17^i^	72.1 (5)	C4—C3—C2	117.7 (13)
O22—Si1—O17^i^	107.9 (5)	C4—C3—H3	121.2
O12^i^—Si1—O17^i^	74.2 (5)	C2—C3—H3	121.2
O12—Si1—O17^i^	105.8 (5)	C5—C4—C3	121.3 (14)
O13—Si1—O17	110.5 (5)	C5—C4—H4	119.4
O13^i^—Si1—O17	69.5 (5)	C3—C4—H4	119.4
O22^i^—Si1—O17	107.9 (5)	N1—C5—C4	122.2 (12)
O22—Si1—O17	72.1 (5)	N1—C5—C6	114.7 (9)
O12^i^—Si1—O17	105.8 (5)	C4—C5—C6	123.1 (12)
O12—Si1—O17	74.2 (5)	N2—C6—C7	109.1 (11)
O17^i^—Si1—O17	180.0 (9)	N2—C6—C5	119.2 (10)
N2—Mn8—N8	98.0 (4)	C7—C6—C5	131.5 (11)
N2—Mn8—N5	95.6 (3)	C8—C7—C6	107.0 (12)
N8—Mn8—N5	161.1 (3)	C8—C7—H7	126.5
N2—Mn8—N7	168.5 (3)	C6—C7—H7	126.5
N8—Mn8—N7	73.2 (4)	C7—C8—N3	106.5 (12)
N5—Mn8—N7	94.8 (3)	C7—C8—H8	126.8
N2—Mn8—N4	89.4 (3)	N3—C8—H8	126.8
N8—Mn8—N4	93.4 (3)	N7—C9—C10	120.7 (11)
N5—Mn8—N4	73.6 (3)	N7—C9—H9	119.6
N7—Mn8—N4	98.3 (3)	C10—C9—H9	119.6
N2—Mn8—N1	73.3 (3)	C11—C10—C9	122.4 (15)
N8—Mn8—N1	100.0 (3)	C11—C10—H10	118.8
N5—Mn8—N1	96.5 (3)	C9—C10—H10	118.8
N7—Mn8—N1	100.6 (3)	C12—C11—C10	120.1 (19)
N4—Mn8—N1	159.4 (3)	C12—C11—H11	119.9
O6—Mo1—O10	103.1 (4)	C10—C11—H11	119.9
O6—Mo1—O7	100.4 (4)	C11—C12—C13	119.2 (16)
O10—Mo1—O7	94.4 (4)	C11—C12—H12	120.4
O6—Mo1—O14	100.4 (4)	C13—C12—H12	120.4
O10—Mo1—O14	89.9 (3)	N7—C13—C12	120.4 (14)
O7—Mo1—O14	157.2 (4)	N7—C13—C14	120.9 (11)
O6—Mo1—O5	97.9 (4)	C12—C13—C14	118.7 (13)
O10—Mo1—O5	158.4 (4)	N8—C14—C15	112.1 (13)
O7—Mo1—O5	86.8 (3)	N8—C14—C13	115.1 (10)
O14—Mo1—O5	81.2 (3)	C15—C14—C13	132.8 (14)
O6—Mo1—O22	159.5 (4)	C14—C15—C16	102.9 (13)
O10—Mo1—O22	66.3 (4)	C14—C15—H15	128.6
O7—Mo1—O22	98.0 (4)	C16—C15—H15	128.6
O14—Mo1—O22	63.4 (4)	N9—C16—C15	104.9 (12)
O5—Mo1—O22	92.1 (4)	N9—C16—H16	127.6
O6—Mo1—O13	155.9 (4)	C15—C16—H16	127.5
O10—Mo1—O13	97.4 (4)	N4—C17—C18	123.3 (10)
O7—Mo1—O13	65.2 (4)	N4—C17—H17	118.4
O14—Mo1—O13	92.0 (4)	C18—C17—H17	118.4
O5—Mo1—O13	63.5 (4)	C17—C18—C19	119.1 (10)
O22—Mo1—O13	43.6 (4)	C17—C18—H18	120.5
O3—Mo2—O5	102.9 (4)	C19—C18—H18	120.5
O3—Mo2—O2	100.6 (5)	C20—C19—C18	118.2 (10)
O5—Mo2—O2	93.5 (4)	C20—C19—H19	120.9
O3—Mo2—O4	98.9 (4)	C18—C19—H19	120.9
O5—Mo2—O4	90.8 (3)	C19—C20—C21	120.8 (10)
O2—Mo2—O4	158.6 (5)	C19—C20—H20	119.6
O3—Mo2—O1	97.9 (4)	C21—C20—H20	119.6
O5—Mo2—O1	158.9 (5)	N4—C21—C20	119.6 (9)
O2—Mo2—O1	85.5 (3)	N4—C21—C22	117.2 (9)
O4—Mo2—O1	83.1 (4)	C20—C21—C22	123.2 (9)
O3—Mo2—O17^i^	154.7 (4)	N5—C22—C23	109.6 (9)
O5—Mo2—O17^i^	98.5 (4)	N5—C22—C21	119.9 (9)
O2—Mo2—O17^i^	64.5 (4)	C23—C22—C21	130.5 (9)
O4—Mo2—O17^i^	94.2 (4)	C24—C23—C22	104.8 (10)
O1—Mo2—O17^i^	62.1 (4)	C24—C23—H23	127.6
O3—Mo2—O13	158.8 (4)	C22—C23—H23	127.6
O5—Mo2—O13	65.2 (4)	N6—C24—C23	107.8 (11)
O2—Mo2—O13	97.7 (5)	N6—C24—H24	126.1
O4—Mo2—O13	65.3 (4)	C23—C24—H24	126.1
O1—Mo2—O13	94.1 (5)	C5—N1—C1	117.1 (10)
O17^i^—Mo2—O13	45.8 (4)	C5—N1—Mn8	116.6 (7)
O19—Mo3—O1^i^	102.9 (5)	C1—N1—Mn8	126.3 (8)
O19—Mo3—O18	101.6 (4)	C6—N2—N3	106.0 (9)
O1^i^—Mo3—O18	95.3 (4)	C6—N2—Mn8	115.9 (7)
O19—Mo3—O20	97.4 (4)	N3—N2—Mn8	138.0 (7)
O1^i^—Mo3—O20	90.5 (4)	N2—N3—C8	111.4 (10)
O18—Mo3—O20	158.4 (4)	N2—N3—H3A	114 (7)
O19—Mo3—O10	96.5 (4)	C8—N3—H3A	133 (6)
O1^i^—Mo3—O10	159.6 (5)	C17—N4—C21	119.0 (8)
O18—Mo3—O10	86.6 (3)	C17—N4—Mn8	126.4 (6)
O20—Mo3—O10	80.9 (4)	C21—N4—Mn8	114.5 (6)
O19—Mo3—O17	157.3 (4)	C22—N5—N6	106.6 (8)
O1^i^—Mo3—O17	64.5 (4)	C22—N5—Mn8	114.5 (7)
O18—Mo3—O17	98.6 (4)	N6—N5—Mn8	138.6 (7)
O20—Mo3—O17	65.3 (4)	C24—N6—N5	111.1 (9)
O10—Mo3—O17	95.1 (4)	C24—N6—H6	124.4
O19—Mo3—O22	153.8 (4)	N5—N6—H6	124.4
O1^i^—Mo3—O22	100.8 (5)	C13—N7—C9	117.1 (11)
O18—Mo3—O22	65.0 (4)	C13—N7—Mn8	113.4 (9)
O20—Mo3—O22	93.4 (4)	C9—N7—Mn8	128.6 (8)
O10—Mo3—O22	61.6 (3)	C14—N8—N9	107.8 (10)
O17—Mo3—O22	47.5 (4)	C14—N8—Mn8	116.6 (8)
O8—Mo4—O11	102.2 (5)	N9—N8—Mn8	135.6 (9)
O8—Mo4—O4	101.5 (4)	N8—N9—C16	112.4 (13)
O11—Mo4—O4	91.5 (4)	N8—N9—H9A	123.8
O8—Mo4—O7	98.4 (4)	C16—N9—H9A	123.8
O11—Mo4—O7	159.2 (5)	Mo3^i^—O1—Mo2	136.1 (6)
O4—Mo4—O7	86.8 (3)	Mo2—O2—Mo6^i^	137.5 (7)
O8—Mo4—O9	99.1 (4)	Mo4—O4—Mo2	135.7 (5)
O11—Mo4—O9	89.7 (3)	Mo2—O5—Mo1	136.4 (5)
O4—Mo4—O9	158.6 (4)	Mo1—O7—Mo4	137.1 (5)
O7—Mo4—O9	84.6 (3)	Mo6—O9—Mo4	136.7 (5)
O8—Mo4—O12	157.2 (4)	Mo1—O10—Mo3	135.9 (5)
O11—Mo4—O12	63.8 (4)	Mo4—O11—Mo5^i^	136.0 (6)
O4—Mo4—O12	96.9 (4)	Si1—O12—Mo4	123.5 (6)
O7—Mo4—O12	95.9 (4)	Si1—O12—Mo5^i^	121.8 (5)
O9—Mo4—O12	64.6 (4)	Mo4—O12—Mo5^i^	97.0 (4)
O8—Mo4—O13	157.1 (4)	Si1—O12—Mo6	118.3 (5)
O11—Mo4—O13	97.1 (4)	Mo4—O12—Mo6	95.3 (4)
O4—Mo4—O13	65.4 (3)	Mo5^i^—O12—Mo6	94.2 (4)
O7—Mo4—O13	63.4 (4)	Si1—O13—O22	57.3 (5)
O9—Mo4—O13	93.2 (4)	Si1—O13—Mo1	123.8 (6)
O12—Mo4—O13	45.3 (3)	O22—O13—Mo1	66.6 (5)
O16—Mo5—O15	101.9 (5)	Si1—O13—Mo2	122.3 (5)
O16—Mo5—O14	101.2 (4)	O22—O13—Mo2	128.5 (6)
O15—Mo5—O14	92.2 (4)	Mo1—O13—Mo2	94.2 (3)
O16—Mo5—O11^i^	100.0 (4)	Si1—O13—Mo4	121.4 (5)
O15—Mo5—O11^i^	88.8 (3)	O22—O13—Mo4	133.5 (6)
O14—Mo5—O11^i^	158.1 (5)	Mo1—O13—Mo4	94.2 (4)
O16—Mo5—O18	100.3 (4)	Mo2—O13—Mo4	93.2 (4)
O15—Mo5—O18	157.4 (5)	Mo5—O14—Mo1	137.5 (5)
O14—Mo5—O18	87.7 (3)	Mo5—O15—Mo6^i^	140.1 (6)
O11^i^—Mo5—O18	83.2 (4)	Si1—O17—Mo3	121.1 (5)
O16—Mo5—O12^i^	156.5 (4)	Si1—O17—Mo2^i^	122.3 (5)
O15—Mo5—O12^i^	64.4 (4)	Mo3—O17—Mo2^i^	97.1 (4)
O14—Mo5—O12^i^	98.4 (4)	Si1—O17—Mo6	119.0 (5)
O11^i^—Mo5—O12^i^	62.5 (4)	Mo3—O17—Mo6	95.3 (4)
O18—Mo5—O12^i^	93.3 (4)	Mo2^i^—O17—Mo6	95.8 (4)
O16—Mo5—O22	156.6 (4)	Mo3—O18—Mo5	137.9 (5)
O15—Mo5—O22	96.0 (5)	Mo6—O20—Mo3	133.8 (5)
O14—Mo5—O22	62.9 (4)	Si1—O22—O13	56.2 (5)
O11^i^—Mo5—O22	95.3 (4)	Si1—O22—Mo1	125.9 (6)
O18—Mo5—O22	63.9 (4)	O13—O22—Mo1	69.8 (5)
O12^i^—Mo5—O22	46.6 (4)	Si1—O22—Mo5	120.2 (5)
O21—Mo6—O20	101.5 (4)	O13—O22—Mo5	129.6 (6)
O21—Mo6—O9	100.2 (4)	Mo1—O22—Mo5	95.5 (4)
O20—Mo6—O9	90.4 (4)	Si1—O22—Mo3	119.1 (5)
O21—Mo6—O2^i^	100.6 (4)	O13—O22—Mo3	134.8 (6)
O20—Mo6—O2^i^	88.9 (3)	Mo1—O22—Mo3	95.7 (3)
O9—Mo6—O2^i^	158.9 (5)	Mo5—O22—Mo3	93.1 (4)
O21—Mo6—O15^i^	100.9 (4)	H1W—O1W—H2W	114 (9)
O20—Mo6—O15^i^	157.5 (5)	H3W—O2W—H3WB	139.00
O9—Mo6—O15^i^	87.5 (3)	H5W—O3W—H6W	114 (9)
O2^i^—Mo6—O15^i^	85.1 (4)		

Symmetry codes: (i) −*x*+1/2, −*y*+1/2, −*z*.

### Hydrogen-bond geometry (Å, °)

**Table:**

*D*—H···*A*	*D*—H	H···*A*	*D*···*A*	*D*—H···*A*
N6—H6···O20	0.86	1.97	2.812 (12)	165
O1W—H1W···O2W	0.82 (5)	1.94 (3)	2.76 (3)	174 (12)
N9—H9A···O3W^ii^	0.86	2.13	2.98 (2)	170
O3W—H5W···O3^iii^	0.82 (6)	2.16 (4)	2.937 (15)	157 (10)
O2W—H3W···O11^iv^	0.82 (13)	2.48 (12)	3.046 (19)	127 (12)
O2W—H3WB···O16^v^	0.9 (6)	2.4 (4)	3.14 (2)	158

Symmetry codes: (ii) *x*−1/2, −*y*+1/2, *z*−1/2; (iii) *x*, *y*, *z*+1; (iv) −*x*+1, *y*, −*z*+1/2; (v) −*x*+1/2, *y*−1/2, −*z*+1/2.
